# Effectiveness of Surface Cleaning and Disinfection in a Brazilian Healthcare Facility

**DOI:** 10.2174/1874434601812010036

**Published:** 2018-03-28

**Authors:** Aires G. Santos-Junior, Adriano M. Ferreira, Oleci P. Frota, Marcelo A. Rigotti, Larissa da S. Barcelos, Alvaro Francisco Lopes de Sousa, Denise de Andrade, Odanir G. Guerra, Mara C. R. Furlan

**Affiliations:** 1 Course of Nursing, Federal University of Mato Grosso do Sul, Coxim, Brazil; 2 School of Medicine, Program of Health and Development in the Center-West Region and Master’s Degree Program in Nursing, Federal University of Mato Grosso do Sul, Campo Grande, Brazil; 3 Course of Nursing, Federal University of Mato Grosso do Sul, Campo Grande, Brazil; 4 Course of Nursing, Federal University of Mato Grosso do Sul, Três Lagoas, Brazil; 5 Department of General and Specialized Nursing, University of São Paulo, Ribeirão Preto School of Nursing, Ribeirão Preto, São Paulo, Brazil

**Keywords:** Surface cleaning, Hospitals, Disinfection, Adenosine triphosphate, Health facility environment, *Staphylococcus aureus*

## Abstract

**Background:**

Failures in the processes of cleaning and disinfecting health service surfaces may result in the spread and transfer of pathogens that are often associated with healthcare-related infections and outbreaks.

**Aims:**

To assess the effectiveness of environmental surface cleaning and disinfection in a hospital clinic.

**Method:**

The study was conducted in a nursing ward with 45 beds. A total of 80 samples from five high-touch surfaces were evaluated before and after cleaning and disinfection, using the following methods: visual inspection, adenosine triphosphate bioluminescence assay, aerobic colony count, *Staphylococcus aureus* colony count, and evaluation of resistance to methicillin. The data analysis used nonparametric comparative and correlative tests to observe any differences in the pre- and post- cleaning and disinfection results for the surfaces assessed.

**Results:**

Effective cleaning and disinfection had a significant effect on only two surfaces when measured for the presence of adenosine triphosphate, the inner bathroom door handle (*p*=0.007) and the toilet bowl (*p*=0.01). When evaluated for *Staphylococcus aureus* colony count, the toilet flush handle also demonstrated a significant effect (*p*=0.04).

**Conclusion:**

The effectiveness of cleaning and disinfection of the surfaces tested was not satisfactory. An educational intervention is recommended for the cleaning and disinfection staff and the nursing team at the healthcare facility.

**Relevance to Clinical Practice:**

The data in the study revealed that daily hospital cleaning and disinfection in the sampled sites are not sufficient in medical and surgical wards. Hospital cleanliness must be reevaluated from the point of view of materials, such as an adequate supply of clean cloths, in addition to establishing more precise cleanliness protocols and accurate monitoring systems.

## 1 INTRODUCTION

Cleaning and disinfection of surfaces in health care are strategies to minimize the occurrence of healthcare-associated infections (HAIs) [1-3]. Considering that the environment serves as a reservoir for microorganisms, even contributing to the transmission of epidemiologically significant pathogens such as methicillin-resistant *Staphylococcus aureus* (MRSA), vancomycin-resistant *Enterococcus* (VRE), *Acinetobacter* spp., *Clostridium difficile* and norovirus [3-5], there is no doubt that containment measures are necessary to break the epidemiological chain of these microorganisms.

For effective infection control, it is recommended that the cleaning and disinfection process should be intensified on high-touch surfaces close to patients, since these surfaces contribute to the transmission of pathogens through hand contamination of health professionals who afterwards come into contact with patients. Examples of these surfaces are: toilets, door handles, bedside tables, telephones, nurse call buttons, and patient chairs, *etc* [5].

Therefore, it is essential to monitor surface cleaning and disinfection. Several methods have been proposed and are widely used to perform these evaluations, such as adenosine triphosphate (ATP) bioluminescence assay and microbiological screening [6]. Both have previously been investigated and recommended as scientific methods for the assessment of the cleanliness of hospital surfaces using ATP reference values, measured in relative light units (RLU), and aerobic colony counts (ACC), measured by colony-forming units per square centimeter (CFU/cm^2^) [7-9].

The objective of the present study was to evaluate the effectiveness of environmental surface cleaning and disinfection in a hospital clinic.

## 2 MATERIALS AND METHODS

### 2.1 Ethical Aspects

This study was approved by the Ethics Committee for Research Involving Human Subjects of the Federal University of Mato Grosso do Sul, Brazil and was carried out in compliance with national and international ethical standards for research.

### 2.2 Design, Location and Time Period

This was a prospective study that was conducted in August 2014 in an internal medicine and surgical nursing ward with 45 beds in a hospital facility located in the state of Mato Grosso do Sul, Brazil. The hospital is a medium-complexity reference for an estimated population of 274,111 residents of the area who use the Unified Health System.

### 2.3 Samples

Non-probabilistic convenience sampling was used; only surfaces frequently touched by patients and professionals from the healthcare facility were considered eligible, since they constitute a potential risk for cross-transmission of microorganisms [10]. The five surfaces selected for the study were: bedside structure (bed rails), bedside table, inner bathroom door handle, toilet bowl rim and toilet flush handle.

Only rooms and beds occupied by patients, with or without HAIs, for over 72 hours were listed for randomization. Samples were collected using the following methods: visual inspection, ATP bioluminescence, ACC, and *Staphylococcus aureus* (*S. aureus*) counts. For surfaces with positive results for *S. aureus*, resistance to methicillin was tested.

The data collection took place in August 2014 during the morning shift, between 7 am and 10 am, twice a week, for four weeks, in one room per collection day, on five surfaces before and after cleaning and disinfection. This generated ten samples for each method per collection day, totaling 80 samples per method at the end of four weeks. The collections were performed before the cleaning staff came to the selected rooms to carry out surface cleaning procedures and after they performed cleaning and disinfection, with a wait of ten minutes for the disinfectant to dry before collecting the samples [3, 9]. Randomization software (http://www.randomization.com) was used for the choice of the days of the week and rooms for collection of samples.

During the study period, no changes were made to the cleaning and disinfection practices for beds or HAI control practices. According to the protocol of the hospital facility, concurrent cleaning was performed once a day in the morning. In addition, the number of beds occupied by patients varied throughout the study.

The disinfectant used was quaternary ammonium-based in combination with polymeric biguanide, called NIPPO-BAC PLUS (Nippon Chemical Ind. e Com. de San. e Det. Prof. Ltda, Brazil). It has an effective property of combining cleaning and disinfection in one step. The surfaces were rubbed randomly with 100% cotton cloths that had been soaked in a previously diluted disinfectant solution. The facility had a standard operational procedure for cleaning and disinfection, in relation to the frequency of replacement of cloths per room, and the study kept the facility’s protocol standard.

### 2.4 Study Protocol

The tests used to monitor cleaning and disinfection were the same as those used in a previous study [3], with the addition of ACC, collected through *RODAC PLATE*^®^ (Replicate Organism Detection and Counting) contact plates, containing tryptone soy agar with neutralizers that inhibit various disinfectants. The collections were taken in a 24 cm^2^ area immediately adjacent to the ATP bioluminescence mold, on both the right and left; the plates were pressed on the surfaces for ten seconds with no lateral movement. They were then incubated at 37 ºC for 24 to 48 hours [11-13]. An electronic digital colony counter (Logen^®^ LS6000) was used for ACC.

Based on previous studies [1-3, 6, 8-13], surface cleaning and disinfection monitoring parameters were established for different methods: ATP bioluminescence (≤250 RLU – acceptable, ^3^250 RLU – unacceptable); ACC (≤2.5 CFU/cm^2^ – acceptable, 2.5 CFU/cm^2^ – unacceptable); and *Staphylococcus aureus*/MRSA (<1 CFU/cm^2^ – acceptable, <1 CFU/cm^2^ - /cm^2^ – unacceptable). In the visual inspection (the first method applied), the surfaces were considered unclean if there was any dust, waste (blood, wound exudates, organic liquids, physiological saline crystals, ointments/creams, oils, solutes, *etc*.), humidity, spots, scratches, cracks or peeling [1, 3].

Susceptibility to methicillin was verified through the triage test for oxacillin resistance. Petri plates were used, containing Muller-Hinton agar supplemented with 4% of NaCl and 6 μg of oxacillin, known as MRSA medium (Probac do Brasil^®^). The microorganisms were transferred to BHI broth and incubated at 37º C for 48 hours. After this period, they were inoculated on plates and incubated at 37º C for 48 hours. Any growth on the plates was considered MRSA.

### 2.5 Results and Statistical Analysis

The collected data was transferred to Minitab 17 (Minitab Inc.) and Statistica 10 (StatSoft Inc) software. The data analysis used nonparametric comparative and correlative tests to observe any differences in the pre- and post- cleaning and disinfection results for the surfaces assessed. Analyses with *p*-values <0.05 were considered significant.

## 3 RESULT

There was a total of 80 surface assessments - 40 before cleaning and disinfection and 40 after. The number and percentage of unapproved surfaces, according to the different methods, varied considerably, as shown in Fig. (**1**).

In Fig. (**1**), the fill patterns of the columns indicate the assessment methods; black is before and gray is after cleaning and disinfection. The results show the proportions found on each of the tested surfaces. Note that the proportions in relation to the MRSA test are in reference to surfaces that tested positive for the presence of *Staphylococcus aureus*. The proportions of unapproved surfaces (showing no improvement in sanitary conditions) before and after disinfection, as measured by visual inspection and positive results of MSRA testing, did not differ significantly.

It is important to note that the ATP readings, expressed in RLU, that were obtained before and after cleaning and disinfection of the five surfaces varied considerably, as demonstrated in Table (**1**).

The RLU median after cleaning and disinfection was less than that obtained before. However, only the bathroom door handle and toilet bowl medians showed statistically significant differences, with *p*=0.007 and *p*=0.100, respectively. Of the surfaces tested, the toilet flush handle was the most unclean, with a median of 176 RLU after cleaning and disinfection.

With respect to ACC on the side of the bed and bedside table, the microbial load was higher after cleaning and disinfection of these surfaces, with medians of 35 and 55.5 before and 59.5 and 71 after cleaning and disinfection, respectively. The CFU count dropped on the other surfaces, but there were no statistically significant differences for any of the surfaces. The toilet bowl was the most unclean surface after cleaning and disinfection, with a median of 157.5 CFU.

In terms of *Staphylococcus aureus* colony count, CFU dropped overall after cleaning and disinfection, but only the bathroom door handle showed a statistically significant difference(p=0.04), as shown in Table (**2**). On the surfaces, the toilet bowl was the least clean after cleaning and disinfection, with a median of 16 CFU of *Staphylococcus aureus.*

Cleaning and disinfection had a significant effect in three situations. The ATP measurement showed statistically significant, lower failure rates after cleaning for the inner bathroom door handle (*p*=0.007) and toilet bowl (*p*=0.01). The *S*. *aureus* count for the toilet flush handle showed a statistically significant difference (*p*=0.04) after cleaning and disinfection. There were no statistically significant differences after cleaning and disinfection for the other surfaces tested. However, the results indicated that the procedure generally resulted in lower microbial loads and ATP readings.

Methicillin resistance was tested for surfaces that had *Staphylococcus aureus* colonies. Before cleaning and disinfection, MRSA was found in one (16.7%) out of six bedside structures, two (40%) out of five bedside tables, three (42.9%) out of seven bathroom door handles, none of the seven toilet bowls, and one (12.5%) out of the eight toilet flush handles. The positive samples after cleaning and disinfection were one (25%) out of four bedside structures, none of the three bedside tables, one (20%) out of five bathroom door handles, one (12.5%) out of eight toilet bowls, and none of the eight toilet flush handles. Therefore, seven (21.5%) of the microbiological samples tested positive for MRSA before cleaning and disinfection, and three (10.7%) of the 28 samples tested positive for MRSA after cleaning and disinfection .

Spearman correlation coefficients were calculated between the results for ATP bioluminescence, *S. aureus* count, and ACC, before and after cleaning and disinfection. The results showed that there were correlations between the methods used in the study. However, the correlations were weak and were only significant between the *S. aureus* count and ACC for the following surfaces: bedside tables (p=0.64, p=0.008), bathroom door handles (p=0.576, p=0.019) and toilet flush handles (p=0.51, p=0.044). There was no significant correlation between the ATP measurement and *S. aureus* colony count or between the ATP measurement and ACC.

## 4 DISCUSSION

In this study, cleaning and disinfection of the studied surfaces showed little effectiveness when assessed by measurement of the presence of ATP and counting colonies of *Staphylococcus aureus*. These findings were in agreement with studies that have shown deficient cleaning in patient units of hospital facilities [3, 8, 14].

Regarding the methods studied, it is noteworthy that, similar to the studied facility, many hospitals still keep visual inspection as the only monitoring method for the cleaning and disinfection process. It is known that hospital surfaces can remain contaminated, mainly by resistant microorganisms, even after the cleaning and disinfection process, if it is unsatisfactory, as evidenced in the present study. Thus, it is relevant to use varied assessment methods for the cleaning and disinfection process, as well as evaluate the correlation among these assessment methods [8, 14, 15].

In the healthcare facility of the present study, the failure rates for visual inspection before and after cleaning and disinfection were higher than those in other studies [1, 3]. However, these rates cannot be solely related to the cleaning and disinfection process; aspects related to deterioration of the surfaces tested need to be taken into account, since most had scratches, cracks, peeling paint, ink stains and glue. If the surfaces tested had not had these structural problems, the results might not have differed so much in relation to other studies.

It is interesting to consider another study that assessed daily surface cleaning practices, using the ATP method, in a university hospital [16]. That study suggested that visual inspection is not sufficient to ensure the quality of the process, and that cleaning levels must be documented through quantitative methods.

Cleaning/disinfection often has effectiveness rates below what would be considered ideal, which demonstrates the need to implement systems to monitor adherence to recommended cleaning practices. This would ensure the quality of hospital surface cleaning and disinfection processes. Another strategy to improve these rates would be monitoring and immediate feedback to the teams responsible for environmental surface cleaning [13-16]. Unfortunately, in the sector evaluated, no type of cleaning and disinfection monitoring process was noted during the data collection period, which could account for the findings. It is also worth noting that in the studied facility, although there was a defined standard operational procedure, low professional adherence was noted, since the frequency of replacement of cleaning cloths and the duration and intensity of the friction applied depended on the professionals; there was no systematic monitoring or assessment.

Unlike other qualitative analysis methods, ATP measurement has been cited as an important tool in cleaning process audits since, unlike visual inspection, it is not subjective. It has also the advantage of providing instant results, unlike microbiological tests, which require 24 to 48 hours to obtain results [1, 8, 9, 17, 18]. ATP analysis evaluates the presence of microbiological and non-microbiological sources, which may be removed by effective cleaning and disinfection protocols. The test can be used to provide immediate surface cleaning and disinfection data, which can be used to demonstrate deficiencies in cleaning and disinfection routines or techniques, evaluate protocols, and train cleaning staff [8, 17].

A study [6] that conducted a cleaning effectiveness audit in four hospitals in the United Kingdom used a set of monitoring methods, including visual inspection, ATP measurement and microbiological analysis. The assessments were done immediately after terminal cleaning in pre-established locations (kitchenettes, bathrooms and beds) of pediatric and surgical nursing wards. The visual inspection indicated that 90% of the surfaces in surgical nursing wards and 100% of the surfaces in pediatric nursing wards were considered acceptably clean. However, the ATP results indicated that none of the surfaces either nursing ward could be considered clean. In the microbiological analysis, monitoring showed that only 10% of the surfaces were acceptably clean.

Significant differences in cleaning and disinfection according to the assessment method were found in the present study. These findings suggest that there are undesirable discrepancies in cleaning and disinfection procedures, in disagreement with the standard operating procedure of the institution, which requires that all surfaces to be cleaned in an identical manner. Therefore, it is reasonable to consider that these differences are due to cleaning operations that are not uniform or standardized. In this regard, cleaning and disinfection monitoring methods may be a quick and effective strategy for detecting variations in cleaning effectiveness over the course of time and checking whether cleaning team members are complying with standardized procedures.

The lack of large enough surface cleaning and disinfection teams is a reality in the hospital studied. The sector has 14 beds, and only two professionals are responsible for daily cleaning of the sector and other locations. It is worth noting that the institution's standard operating procedure delegates surface cleaning and disinfection to the hospital cleaning staff; the nurses have no responsibility in this regard. The result is probably that the main concern of the cleaning staff is the amount of time spent on cleaning, not the quality of the process performed [9].

The authors agree with the U.S. Centers for Disease Control and Prevention, which argues that health professionals, i.e., nursing teams, are responsible for acting as supporting professionals and paying special attention to ensuring adequate cleaning and disinfection of the units where they work, since they are indispensable professionals who collaborate with cleaning services and infection control programs [18].

Another study carried out in a Brazilian intensive care unit [15] over a period of 14 days sought, through three monitoring methods, to determine the cleaning and disinfection conditions of four surfaces close to patients (bed rails, cranks, bedside tables and infusion pump buttons) after a concurrent cleaning process with 70% isopropyl alcohol. Out of a total of 100 samples collected, 20%, 80% and 16% of the assessments by visual inspection, ATP, and presence of *Staphylococcus aureus*/MSRA, respectively, were considered as unapproved. There were statistically significant differences (*p*<0.05) among the unapproved cleaning rates according to ATP compared with visual inspection and microbiological methods. There was no correlation between the results for ATP and *Staphylococcus aureus*/MRSA, which supports the findings of the present study.

Another study demonstrated that surface cleaning and disinfection monitoring using fluorescent gel markers or ATP were more accurate than visual inspection. It was noted that of the 250 surfaces tested, 214 (86%) had no visible contamination before cleaning. The rate rose to 232 (93%) after the cleaning and disinfection process. However, of the same surfaces tested using ATP count, only 132 (53%) were considered clean before terminal cleaning, which increased to 191 (76%) after. The same occurred with the aerobic culture, in which 148 (59%) were considered clean before and 218 (87%) after cleaning [19].

Use of cotton cloths may have had a bearing on the low cleaning and disinfection rates in the current study, as corroborated by a recent study that compared the impact of using two types of fabric (cotton and microfiber) on the concentration of three brands of quaternary ammonium. The results showed that the concentration of the tested disinfectant (quaternary ammonium), when exposed to cotton cloths used for surface cleaning and disinfection, can be reduced by up to 85.3%, and was inactivated in over 80% after five minutes of contact. This is due to the high cellulose concentrations in cotton cloths [20].

The current study only evaluated the presence of *S. aureus* and MRSA. Therefore, its findings cannot be generalized for interventions designed to reduce surface contamination by other microorganisms (such as Gram-positive pathogens).

Correlation coefficients were calculated between the ATP (RLU), *S. aureus* count and ACC methods. However, the correlations were low and only significant between the *S. aureus* count and ATP on bedside tables, bathroom door handles and toilet flush handles. There was no significant correlation between the ATP measurement and *S. aureus* colony count or between the ATP measurement and ACC.

Studies that have compared the ATP (RLU) method with microbiological cultures have found, as in the present study, no correlation between the RLU and ACC [3, 15, 17]. However some authors [2, 9, 21] have reported good concordance or weak relationships between the two methods, suggesting that surfaces with low microbial contamination also have low RLU readings, and that RLU may account for around one-third of the variability in ACC [22]. According to this observation, researchers found that approximately 33% of microbial ATP probably originates from hand-touch surfaces and the rest from non-microbial origins [23].

In the present study, no correlations were found between the cleaning and disinfection monitoring methods. In another study [16], critical surfaces classified as “clean” according to fluorescent markers were often also “clean” according to microbiological criteria, whereas this situation was much less often true for ATP bioluminescence. Another study [24] showed no correlations between two quantitative methods (ATP bioluminescence and CFU) and fluorescent markers, i.e., neither of the quantitative methods correlated well with the fluorescent gel results.

Correlations between methods for measuring environmental cleaning have been inconsistent, although they measure different parameters. Further studies are needed to evaluate the correlations and predictive values of the three assessment methods for environmental surface cleaning [24-25].

Although more studies are necessary, the authors believe that each method, whether visual inspection, ATP bioluminescence or microbial count, should not exclude the others, but be used in a complementary manner, since each analysis and quantification sheds light on different aspects of the operations needed to achieve good levels of environmental cleanliness.

The present study had certain limitations. It was conducted in only one institution and in a nursing ward where there was no evaluation of cleaning and disinfection practices during sampling. The sample size was small. The design of the study did not permit establishing a relationship between the results of the methods before and/or after cleaning and disinfection and the risk of acquiring HAIs. From another viewpoint, the methodology used may serve as a reference for healthcare facilities to examine their cleaning and disinfection protocols, in order to identify weak points in the process. Therefore, the present study suggests that it would be useful to investigate best cleaning and disinfection practices in the healthcare field to provide nursing teams and health service users with safer and better-quality care from an environmental cleanliness point of view. Finally, each healthcare facility should evaluate its cleaning and disinfection processes, whether performed by nursing teams or cleaning staff.

## CONCLUSION

The cleaning and disinfection procedures for high-touch surfaces performed in the facility presented flaws in relation to their effectiveness, when they were analyzed by different monitoring methods. Some specific aspects may have influenced these results, such as the age of the furniture assessed and the lack of adoption of practice standardization by the cleaning team.

In the light of the described findings, an educational intervention is recommended for the cleaning and disinfection staff and nursing team of the hospital studied, since this practice helps improve the results of measurements of surface cleaning and disinfection. This leads to a reduction in the risk of patients being affected by colonization and/or healthcare-associated infections from surfaces.

## Figures and Tables

**Fig. (1):**
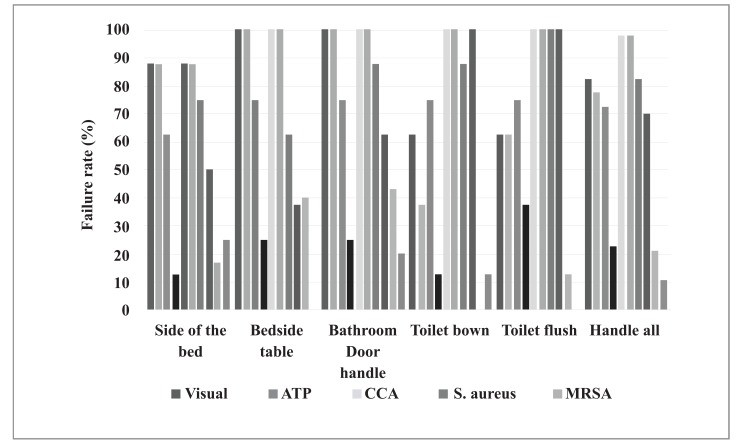
Failure rates before and after cleaning/disinfection. Três Lagoas, MS, Brazil, 2014.

**Table 1:** ATP readings on different surfaces before and after cleaning/disinfection. Três Lagoas, MS, Brazil, 2014.

**Surfaces**	**Time of cleaning**				*P†*
	**Before**		**After**		
	Median (RLU)	Variation (RLU)	Median (RLU)	Variation (RLU)	
Side of the bed (n=16)‡	458	11-3,693	136	22-1,665	0.054
Bedside table (n=16)	654	174-2,479	107	31-3,873	0.147
Bathroom door handle (n=16)	358	173-4,512	137	16-2,044	**0.007**
Toilet bowl (n=16)	758	25-1,117	47	22-551	**0.010**
Toilet flush handle (n=16)	946	107-35,453	176	36-74,791	0.363

*P*† refers to the Wilcoxon signed-rank test at *p*<0.05. ‡ corresponds to the eight surfaces assessed before and after cleaning/disinfection.

**Table 2:** *Staphylococcus aureus* CFU readings on different surfaces before and after cleaning/disinfection. Três Lagoas, MS, Brazil, 2014.

**Surfaces**	**Time of cleaning**				*P†*
	**Before**		**After**		
	Median CFU (*S. aureus*)	Variation CFU (*S. aureus*)	Median CFU (*S. aureus*)	Variation CFU (*S. aureus*)	
Side of the bed (n=16)‡	3	0.0-22	0.5	0.0-360	0.458
Bedside table (n=16)	1	0.0-270	0.0	0.0-11	0.050
Bathroom door handle (n=16)	12	0.0-150	8.5	0.0-49	0.176
Toilet bowl (n=16)	17.5	0.0-330	16	2-35	0.181
Toilet flush handle (n=16)	10.5	2-390	3.5	1-16	**0.040**

*P*† refers to the Wilcoxon signed-rank test at *p*<0.05. ‡ corresponds to the eight surfaces assessed before and after cleaning/disinfection.
